# Morally injurious events among aid workers: examining the indirect effect of negative cognitions and self-care in associations with mental health indicators

**DOI:** 10.3389/fpsyg.2023.1171629

**Published:** 2023-04-13

**Authors:** Michelle Dewar, Alison Paradis, Pascale Brillon

**Affiliations:** ^1^Laboratory for the Study of the Well-Being of Families and Couples, Department of Psychology, Université du Québec à Montréal, Montreal, QC, Canada; ^2^Trauma and Resilience Lab, Department of Psychology, Université du Québec à Montréal, Montreal, QC, Canada

**Keywords:** potentially morally injurious events, depression, PTSD, posttraumatic growth, cognitions, self-care, aid workers

## Abstract

**Introduction:**

Potentially morally injurious events (PMIE) are events that violate one’s deeply held moral values or beliefs, and that have the potential to create significant inner conflict and psychological distress. PMIE have been recognized as an important psychological risk factor in many high-risk occupational groups. However, no study to date has investigated how PMIE relate to the mental health of aid workers. Furthermore, little is known about the mechanisms by which PMIE might be associated with mental health indicators.

**Methods:**

Participants were 243 aid workers (72% female; *M*_age_ = 39.31) who had completed at least one aid assignment (*M* = 8.17). They completed an online questionnaire about their PMIE, trauma history, and mental health. A structural equation model was constructed to examine the roles of negative cognitions and subsequent self-care behaviors in the relationship between PMIE and PTSD symptoms, depression symptoms, and posttraumatic growth, above and beyond the contribution of potentially traumatic events.

**Results:**

Within the model, the indirect effect through negative cognitions fully accounted for the associations between PMIE and symptoms of PTSD and depression. For the association between PMIE and posttraumatic growth, two indirect effects emerged: the first through negative cognitions and subsequent self-care and, the second, through self-care alone.

**Discussion:**

This study highlighted PMIE as a novel psychological risk factor for aid workers and pointed to two possible mechanisms by which these events may lead to PTSD, depression, and posttraumatic growth. This study adds to the current understanding of how high-risk occupational groups adapt psychologically to PMIE.

## 1. Introduction

The nature of their work implies that aid workers frequently encounter human distress and witness numerous injustices that are inherent to humanitarian crises (Stoddard et al., 2014). An increasing number of qualitative studies have suggested that aid workers are exposed to ethical issues and moral conflicts. For instance, in a qualitative case study, researchers found that struggling to “understand global injustices and acts of inhumanity,” to “bring meaning to confronting situations,” and to cope with “a sense of helplessness in horrific situations” were major issues that resulted from the participant’s aid experience (McCormack et al., 2009). Another study found that aid workers who provided healthcare reported severe distress that stemmed from having to choose which seriously ill patients to treat (and which ones not to treat) because of limited resources (Goodman and Black, 2015). Similarly, in Hunt’s (2008) study, aid workers reported a deep sense of unease when accepted practices were contrary to their core values. Indeed, many were torn about whether they should abide by policies they saw as unfair or discriminatory (e.g., based on ethnicity, socioeconomic status, gender). McCormack and Joseph (2013) reported that many aid workers questioned their own actions, particularly when they chose to prioritize their own safety over that of others. Such situations generated a great deal of shame and the fear of being judged. In fact, a literature review found that being confronted with morally challenging situations was one of the major themes emerging from qualitative studies focused on the stressors that aid workers face (Nordahl, 2016). This review suggested that future studies should explore how such stressors might be associated with mental health indicators. Yet, no single study exists that has investigated if and how potentially morally injurious events (PMIE) might relate to the mental health of aid workers.

The term PMIE was coined to designate events can be traumatic because they involve “perpetrating, failing to prevent, bearing witness to, or learning about acts that transgress deeply held moral beliefs and expectations” (Litz et al., 2009). Three main types of PMIE have been documented in the military literature: PMIE that involve one’s own wrongful actions, PMIE where the actions of others are interpreted as wrong (e.g., as witnesses or victims), and lastly, PMIE where one perceives to have been betrayed by a trusted individual or organization (Litz et al., 2009). Because aid workers operate under different circumstances than military populations, it is unclear whether these three types of PMIE will also be reported by aid workers. Understanding if and how aid workers experience PMIE has important implications for the development of measures to prevent them and limit their consequences.

In recent years, research has highlighted just how psychologically damaging PMIE can be, as its deleterious consequences have become clear. One of the core difficulties is the presence of strong unpleasant moral emotions; shame, guilt, helplessness, anger, and betrayal (Yeterian et al., 2019). Other emotions often associated with PMIE include grief, sadness, anxiety, and disgust (Griffin et al., 2019). Those exposed to PMIE also show destructive behavior, suicidal ideation, self-sabotage, reckless behavior, hypervigilance, nightmares, substance use, self-harm, social withdrawal, fear of being misunderstood or rejected by loved ones, interpersonal difficulties, cynicism, loss of religious faith, loss of meaning in life, trust issues, and feelings of being “broken” [see review by Griffin et al. (2019)]. Indeed, a meta-analytic systematic review of studies on the sequelae of PMIE in occupational populations found significant associations with PTSD and depression symptoms (weighted mean Cohen’s effect sizes of, respectively 0.30 and 0.23; Williamson et al., 2018). These disorders are unfortunately prevalent among aid workers. In fact, at any given time, between 6.2 and 43.0% of aid workers reported clinical levels of PTSD (median prevalence in two reviews were of 17.0 and 19.1%; Connorton et al., 2012; Strohmeier and Scholte, 2015), and between 4.0 and 58.0% of clinical depression (median prevalence in two reviews were 18.5 and 27.1%; Connorton et al., 2012; Strohmeier and Scholte, 2015). The significant variations in disorders’ prevalence may be due to the homogeneous samples used in most studies on aid workers (e.g., aid workers only working in Kosovo; Holtz et al., 2002; Cardozo et al., 2005). Differences in the type of assignments or level of security in the geographic region may have contributed to important variations in prevalence (e.g., Solomon and Green, 1992). Nevertheless, a significant proportion of aid workers report PTSD and depression. Further, the consequences of PMIE appear to be quite pervasive, and to contribute to symptoms of PTSD and depression. However, the presence of PMIE among aid workers and its contribution to mental health difficulties remain to be explored in this population.

It is well-known that fear-based traumatic events (those that threaten life, safety, or integrity; known as potentially traumatic events; American Psychiatric Association [APA], 2013) can be detrimental to mental health. However, traumatic events also can positively impact mental health by generating posttraumatic growth [see review by Wu et al. (2019)]. Posttraumatic growth designates the positive psychological changes that can occur in several areas of life as a result of the struggle with difficult or traumatic events (Tedeschi and Calhoun, 2004). In fact, trauma may lead people to question their life objectives, to develop a new understanding of what is important in their lives, to develop a stronger sense of personal strength, to feel that their social relations are more intimate or meaningful, and to have a greater appreciation of life (Tedeschi and Calhoun, 2004; Joseph, 2012). The scant available research seems to support the idea that posttraumatic growth can also occur in the face of PMIE. In one study of veterans, PMIE predicted 30.0% of the variance in posttraumatic growth (β = 0.55, *p* < 0.001, *n* = 120; Breazeale, 2019). In another study of combat veterans and prisoners of war (*n* = 221), guilt-induced distress was associated with more posttraumatic growth both cross-sectionally (*r* = 0.36, *p* < 0.01) and longitudinally (β = 0.32, *p* = 0.02; Dekel et al., 2016). Thus, to adequately capture how PMIE are associated to the mental health of aid workers, it appears essential to consider *positive* indicators such as posttraumatic growth in addition to indicators of psychological distress such as symptoms of PTSD and depression. Furthermore, relatively little is known about the mechanisms by which both distress and growth can occur following PMIE exposure (Griffin et al., 2019; McEwen et al., 2021).

Theoretical models suggest several mechanisms that might contribute to how individuals adapt psychologically to a PMIE, thereby contributing to our understanding of how both posttraumatic growth and psychological distress, namely posttraumatic and depressive symptoms, may emerge (Litz et al., 2009). PMIE represents a significant challenge to previously held beliefs about one’s ability to live up to moral expectations, one’s basic moral worth and the goodness of the world, for example. Because of this incongruence, individuals attempt to integrate the meaning of the PMIE into their preexisting beliefs by interpreting the causes, consequences, and meaning of the events. In this process of reconsidering their fundamental beliefs about who they are, what others are like, what kind of world they live in, or what the future may hold, individuals may develop a sense that the PMIE has led to valuable, though painful, life lessons that give rise to posttraumatic growth (Tedeschi and Calhoun, 2004). In fact, the meaning that is given to the PMIE is proposed to be a determining factor in the positive or negative psychological adaptation that may result. If this incongruence gives rise to stable, internal, and global negative cognitions about themselves (e.g., “I am a bad person”), the cause of events (e.g., “There is something wrong about me for having let this happen”) or the world (e.g., “People are evil”; Foa et al., 1999), these interpretations will lead to long-lasting painful emotions such as guilt, anger, shame, or hopelessness, which are central to PTSD and depression.

These cognitions and painful moral emotions are also reflected in the behaviors that individuals engage in. In fact, one behavioral mechanism that may be important to investigate to understand how aid workers might adapt psychologically to PMIE is self-care. Self-care generally designates behaviors that one implements in several areas of life to get psychological relief (e.g., to reduce depression), maximize their vitality, and be more resilient (e.g., prevent compassion fatigue), thereby promoting a subjective sense of wellbeing (Lee and Miller, 2013; Bloomquist et al., 2015). Examples of self-care might be getting adequate sleep (physical self-care), taking time for reflection (psychological self-care), spending time with people that one enjoys (emotional self-care), spending time with nature (spiritual self-care), or taking breaks during the workday (professional self-care; Bloomquist et al., 2015). Because self-care is a self-initiated behavior, it is viewed as a tool that individuals naturally use to adapt and maximize good psychological adjustment when faced with challenging times (Lee and Miller, 2013). For this reason, investigating if self-care practice relates to mental health indicators in the face of PMIE is particularly pertinent as it allows to explore if self-care might contribute to our understanding of how aid workers adapt to the moral questioning that might result from PMIE. In fact, the behaviors that individuals will adopt in the aftermath of PMIE appear to be largely dependent on how they “make sense” of these experiences (Litz et al., 2009). For instance, an aid worker who believes that “I did my best in difficult circumstances” might increase their self-care, whereas one who comes to believe that “I can do no good in the world” as a result of a PMIE may neglect their own needs and reduce their self-care. Developing a more balanced meaning of the PMIE (e.g., “I wish that it had never happened, but I am still someone who is worth loving”) may promote more adaptive behaviors such as disclosure, seeking out support, taking reparative actions, or engaging in more self-care behaviors. Therefore, we argue that changes in self-care that occur after having experienced a PMIE are secondary to one’s cognitions, and might in part explain symptoms of PTSD, depression, and posttraumatic growth.

### 1.1. Objectives of the present study

**FIGURE 1:**
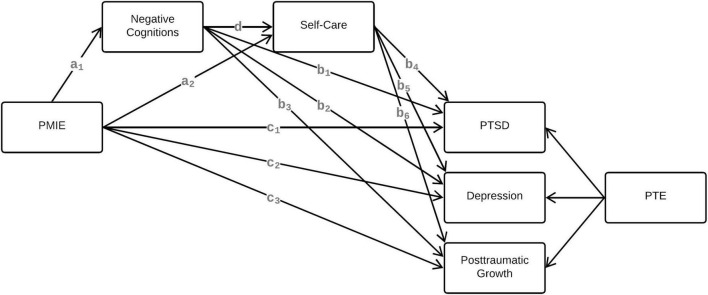
Hypothesized model for the effect of PMIE on PTSD symptoms, depression symptoms, and posttraumatic growth, after accounting for PTE. PMIE, potentially morally injurious events; PTSD, posttraumatic stress disorder; PTE, potentially traumatic events.

The goal of the current study was to (1) examine the presence of PMIE in aid workers, and (2) understand how they were associated with PTSD symptoms, depression symptoms, and posttraumatic growth in this population. Furthermore, there is a lack of evidence pertaining to the mechanism explaining the relationship between PMIE and mental health indicators (Griffin et al., 2019; McEwen et al., 2021). Thus this study aimed (3) to examine if negative cognitions and self-care behaviors might contribute to the associations between PMIE and PTSD symptoms, depression symptoms, and posttraumatic growth, above and beyond the contribution of potentially traumatic events. The proposed model is presented in Figure 1. We wanted to test whether the hypothesized positive associations between PMIE and PTSD, depression, and posttraumatic growth in aid workers could be explained, at least in part, by the negative cognitions and self-care behaviors that they engage in, regardless of how many potentially traumatic events they had experienced. Developing a stronger understanding of these two potential mechanisms will help researchers develop better interventions that build off mechanisms that promote growth and wellbeing while targeting others that exacerbate distress.

## 2. Materials and methods

### 2.1. Sample and procedure

The recruitment of participants was predominantly carried out via aid organizations of various sizes (both major and smaller aid organizations) from the public, non-profit and private sector. Organizations that agreed to participate sent an email invitation for the study to their staff directly. The invitation text and flyer were provided and clearly indicated that the study was confidential and anonymous. This procedure was selected to respect the organizations’ obligation to protect their staff’s privacy. Participants were also recruited through targeted posts on social media groups for aid workers. These invitations contained a link to the *Qualtrics* web platform where they completed a written consent form and the survey. To participate, aid workers had to have a sufficient level of English or French, have completed at least one aid assignment, and be at least 18 years of age. Participants were not compensated for their participation. Ethical approval for the current study was obtained from the University of Quebec in Montreal Institutional Ethics Committee on Human Research.

The sample consisted of 243 aid workers who were 39.31 years of age on average (*SD* = 10.63, range: 23–72). Most participants identified as women (72%) and were highly educated (96.3% had at least completed college, an undergraduate degree, or the equivalent). Aid workers identified as Caucasian (75.8%), Black (11.1%), Middle Eastern (5.3%), Hispanic or Latino/a/x (1.6%), Asian or Pacific Islander (3.7%), Indigenous or First Nations (1.2%), and any other ethnicity or origin (6.6%). Many reported being married, in a civil union, or cohabiting with their partner (44.7%), others reported being single (45.1%), and the rest reported being divorced, widowed, or separated (10.2%). Regarding their work experience, they had completed an average of 8.17 assignments (*SD* = 8.91); 44.7% reported completing only humanitarian aid assignments, 38.9% reported completing both development and humanitarian aid assignments, and 16.4% reported completing only development assignments. During their last assignment, most were expatriated (74.8%). In terms of role, 36.1% worked as team or project managers, 18.9% as health, mental health, or nutrition professionals or technicians, 9.0% did administrative work, 6.6% worked in program evaluation, 6.6% in sanitation, agriculture, or food safety, 5.3% in logistics or infrastructure, 4.5% in legal, advocacy, or human rights, and 13.0% occupied other roles.

### 2.2. Instruments

#### 2.2.1. Potentially morally injurious events

The *Moral Injury Appraisals Scale* (MIAS; Hoffman et al., 2018) measures exposure to potentially morally injurious events across three subscales: the PMIE that involve one’s own actions subscale (five items; α = 0.88; e.g., “I am disturbed because I have acted contrary to important moral rules”); the PMIE relating to others’ actions subscale (four items; α = 0.87; e.g., “I am disturbed by immoral things that other people have done”); the PMIE betrayal subscale (two items) designates events when they perceive to have been betrayed by someone they trusted or when they betrayed someone who trusted them (e.g., “I betrayed people who trusted me”). Each of the 11 items is rated on a four-point Likert scale (1 = *not at all*; 4 = *very much*). Higher total (α = 0.84) and subscale scores indicate greater PMIE exposure.

#### 2.2.2. PTSD symptoms

The *Posttraumatic Stress Disorder Checklist for DSM-5* (PCL-5; Blevins et al., 2015) was used to assess the intensity of PTSD symptoms (as outlined in the DSM-5; American Psychiatric Association [APA], 2013). This 20-item questionnaire asks participants to indicate how often, in the past month, they have been bothered by each symptom using a five-point Likert scale (0 = never, 4 = extremely). A higher total score, ranging from 0 to 80, is associated with more severe symptoms (α = 0.95). A cut-off of 33 is suggested for probable PTSD diagnosis (Blevins et al., 2015).

#### 2.2.3. Depression symptoms

The *Patient Health Questionnaire* (PHQ-9; Kroenke et al., 2001) assesses symptoms of depression over the past 2 weeks. Participants rated nine items (e.g., feeling down, depressed, or hopeless) on a four-point Likert scale (0 = never, 3 = almost every day). For the total score (possible range: 0–27; α = 0.88), a score of 10 or higher is the threshold for clinical depression symptoms (Kroenke et al., 2001).

#### 2.2.4. Posttraumatic growth

The *Posttraumatic Growth Inventory*-*Short Form* (PTGI-SF; Cann et al., 2010) measures the degree to which participants experience positive changes in their lives in the aftermath of adversity. Participants rated 10 items (e.g., “I established a new path for my life”) on a six-point Likert scale (0 = I did not experience this change to 5 = I experienced this change to a very great degree). We used the total score (possible range: 0–50; α = 87).

#### 2.2.5. Negative trauma-related cognitions

The *Posttraumatic Cognitions Inventory*-9 (PTCI-9; Wells et al., 2019; Lebel et al., 2022) is a nine-item measure of trauma-related negative cognitions that includes three subscales: negative cognitions about the “self” describes a negative view of oneself and a loss of self-trust (e.g., “I feel like I don’t know myself anymore”); negative cognitions about the world focuses on how the world is fundamentally unsafe and others are untrustworthy (e.g., “People can’t be trusted”); lastly, negative cognitions about self-blame suggests that one is to blame for what happened (e.g., “Somebody else would not have gotten into the situation”). Items are rated on a seven-point Likert scale (1 = totally disagree, 7 = totally agree). A higher total score (α = 0.85) indicates more negative cognitions.

#### 2.2.6. Self-care practice

The *Self-Care Practice Scale* (Bloomquist et al., 2015) measured the participants’ self-care practices over the past 30 days. This measure assessed five domains of self-care practice—physical, professional, emotional, psychological, and spiritual, with each domain including eight to 12 self-care behaviors, for a total of 45 items. As this instrument was originally designed for mental health professionals, some of the professional self-care items were adapted to be relevant to the reality of aid workers. For example, “Discuss cases with colleagues” was changed to “Discuss work situations with colleagues.” Participants rated the frequency with which they engaged in each behavior using a six-point Likert scale (0 = never; 6 = very frequently). A higher total score (α = 0.92) indicates more frequent self-care.

#### 2.2.7. Potentially traumatic experiences

The *Life Events Checklist for the DSM–5* (LEC-5; Blevins et al., 2015) assessed lifetime exposure to potentially traumatic events. Two aid-specific items were added to reflect experiences that are common for aid workers (Eriksson et al., 2001)—“Survival-related fear (e.g., not having access to food, water or shelter)” and “Exposure to dead bodies other than during a funeral (e.g., a mass grave)”—for a total of 19 items. Participants indicated if they had experienced each item (1 = yes, 0 = no) in four contexts: (1) happened to me, (2) witnessed it, (3) learned that it happened to a close family member or friend, and (4) happened to me as a part of my job. A higher total score (possible range: 0–76) indicates greater potentially traumatic exposure.

### 2.3. Data analysis plan

**FIGURE 2:**
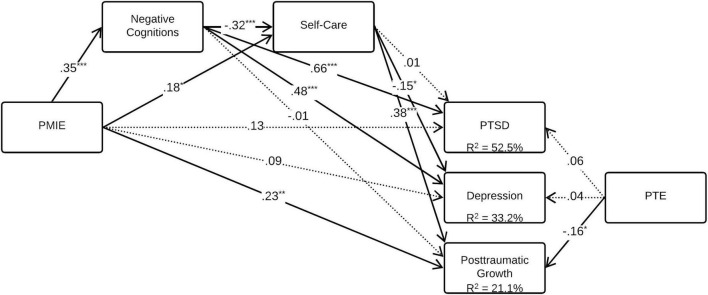
Estimated model for the effect of PMIE on PTSD symptoms, depression symptoms, and posttraumatic growth, after accounting for PTE. All coefficients are standardized. Significant paths are represented by continuous lines and non-significant paths are represented by dotted lines. Numbers under the three mental health indicators indicate the amount of variance explained (R^2^). PMIE, potentially morally injurious events; PTSD, posttraumatic stress disorder; PTE, potentially traumatic events. *p < 0.05, **p < 0.01, ***p < 0.001.

Descriptive statistics, prevalence of PMIE, rates of PTSD and depression, bivariate Pearson’s correlations among study variables, and Cronbach’s α for each of the measures were calculated in SPSS 27. Before running the main model analysis, missing data and assumptions were examined. There was no missing data on the study variables; therefore, the final sample consisted of 243 aid workers. Next, we constructed a structural equation model (see Figure 1) examining the indirect effect of negative cognitions (1st) and subsequent self-care (2nd) in the relationship between PMIE and three mental health indicators: PTSD symptoms, depression symptoms, and posttraumatic growth. To accomplish this, we built a model that included both negative cognitions and self-care and assessed three specific indirect effects, which provided information regarding the role of each variable in the relation between the independent variable and each of the dependent variables: (1) through negative cognitions alone (e.g., path a_1_ → b_1_ in Figure 1), (2) through negative cognitions and self-care in a serial fashion (e.g., path a_1_ → d → b_4_ in Figure 1), and (3) through self-care alone (e.g., path a_2_ → b_4_ in Figure 1). The effect of potentially traumatic exposure on the mental health indicators was entered as a control to make sure that a potential indirect effect would not be artificially inflated by the potentially traumatic events that aid workers may also have experienced. The model was constructed in Mplus 7.4 using structural equation modeling and a ML estimator. The mean scores were used for all variables except for potentially traumatic exposure, for which the total score was used. The statistical significance of each indirect effect was tested with a bias corrected and accelerated 95% confidence intervals (CI) obtained from 10,000 bootstrapped samples. A CI that does not include zero provides evidence of a significant indirect effect (Preacher and Hayes, 2008). Five indices were used to assess this model’s fit: a non-significant Chi-Square (*X*^2^), the Tucker-Lewis index (TLI) and comparative fit index (CFI) > 0.95, the root mean square error approximation (RMSEA) < 0.06, and, lastly, the standardized root mean square residual (SRMR) < 0.09 indicated that the model fit the data well (Hu and Bentler, 1999).

## 3. Results

### 3.1. Prevalence of PMIE, potentially traumatic exposure and levels of mental health indicators

Much of the sample (81.1%) reported exposure to PMIE, with other-related PMIE being much more prevalent (75.3%) than betrayal-related PMIE (27.4%) and self-related PMIE (22.8%). See Supplementary Table 1 for details on PMIE exposure. Participants were also highly exposed to potentially traumatic events; all participants reported experiencing at least one potentially traumatic event, with the mean number of events being 16.39 (*SD* = 13.63). Regarding distress levels and according to recommended cut-offs, 19.3% reported clinical PTSD (Blevins et al., 2015) and 24.2% reported clinical depression (Kroenke et al., 2001); 39.3% reported mild depressive symptoms, 12.7% reported moderate, 7% reported moderately severe, and 4.5% reported severe depression. When considering the co-occurrence of both clinical PTSD and depression, 4.5% of the sample reported clinical PTSD alone, 9.4% reported clinical depression alone, and 14.8% reported both. Finally, the mean score for posttraumatic growth was 36.82 (SD = 10.23) which is considered high. Indeed, in comparison, other high-risk occupational groups such as veterans and firefighters have, respectively reported means of 17.11 (SD = 14.18; *n* = 3,157; Tsai et al., 2015) and 13.23 (SD = 11.13; *n* = 1,916; Kehl et al., 2014) on the same scale (i.e., PTGI-SF).

### 3.2. Relationships between study variables

Table 1 shows the means, standard deviations, and correlations of the study variables. Increasing levels of PMIE were significantly associated with higher PTSD and depression symptoms, posttraumatic growth, and negative cognitions. PMIE was not significantly related with self-care practices.

**TABLE 1:** Descriptive statistics and correlations for study variables.

Variable		*M*	*SD*	1	2	3	4	5	6
1	PMIE^a^	1.94	0.53	–	–	–	–	–	–
2	PTSD	18.21	15.6	0.37***	–	–	–	–	–
3	Depression	7.04	5.51	0.26***	0.73***	–	–	–	–
4	Posttraumatic growth	36.82	10.23	0.22***	0.09	-0.04	–	–	–
5	Negative cognitions^a^	2.61	1.03	0.36***	0.72***	0.59***	-0.06	–	–
6	Self-care^a^	3.35	0.64	0.04	-0.14**	-0.23***	0.39***	-0.26***	–
7	PTE	16.39	13.63	0.23***	0.19**	0.13*	-0.10	0.22***	0.01

*p < 0.05, **p < 0.01, ***p < 0.001. PMIE, potentially morally injurious events; PTSD, posttraumatic stress disorder; PTE, potentially traumatic events.

^a^The mean item ratings are presented for PMIE (range: 1–4), negative cognitions (range: 1–6) and self-care (range: 1–6).

All other scores are mean total score.

### 3.3. Main structural equation model

To test whether negative cognitions and self-care could be mechanisms by which PMIE may be associated to PTSD symptoms, depression symptoms, and posttraumatic growth, we built a structural equation model. For each mental health indicator, we tested for three potential indirect effects: (1) through negative cognitions alone, (2) through negative cognitions and self-care in a serial fashion, and (3) through self-care alone. Several statistically significant indirect paths were found (results are shown in Figure 2 and Table 2). For both associations between PMIE and PTSD and between PMIE depression symptoms, only the indirect effect via negative cognitions was significant (β = 0.23, *p* < 0.001 and β = 0.16, *p* < 0.001, respectively). In fact, the indirect effects via self-care alone, or the compound indirect effect of negative cognitions and self-care were not significant. In contrast, once the indirect effect through negative cognitions was entered, the direct effect was no longer statistically significant for both PTSD and depression symptoms (β = 0.13, *p* = 0.06 and β = 0.09, *p* = 0.22, respectively). Therefore, the more aid workers reported PMIE exposure, the more they reported negative cognitions, which was associated to more depressed and reporting more severe PTSD symptoms.

**TABLE 2:** Total, indirect and direct effects of potentially morally injurious events (PMIE), negative cognitions and self-care on posttraumatic stress disorder (PTSD) symptoms, depression symptoms and posttraumatic growth.

Mental health indicator	PTSD			Depression			Posttraumatic growth		
	β	*SE*	95% CI	β	*SE*	95% CI	β	*SE*	95% CI
Total	0.36***	**0.05**	**(0.27, 0.44)**	0.25***	**0.07**	**(0.14, 0.35)**	0.26***	**0.06**	**(0.15, 0.35)**
Total indirect	0.23***	**0.05**	**(0.15, 0.33)**	0.16***	**0.05**	**(0.08, 0.24)**	0.02	0.04	(−0.05, 0.09)
**Specific indirect**									
Via negative cognitions	0.23***	**0.05**	**(0.15, 0.33)**	0.17***	**0.04**	**(0.11, 0.24)**	−0.001	0.03	(−0.05, 0.04)
Via self-care	0.001	0.01	(−0.01, 0.02)	−0.03	0.02	(−0.07, −0.01)	0.07*	**0.03**	**(0.02, 0.12)**
Via negative cognitions and self-care	−0.001	0.001	(−0.01, 0.01)	0.02	0.01	(0.01, 0.04)	−0.04**	**0.02**	**(−0.08, −0.02)**
Direct	0.13	0.07	(0.02, 0.23)	0.09	0.07	(−0.04, 0.20)	0.23***	**0.07**	**(0.12, 0.33)**

*p < 0.05, **p < 0.01, ***p < 0.001. PMIE, potentially morally injurious events; PTSD, posttraumatic stress disorder. Values in bold are statistically significant at p < 0.05 level.

However, different indirect effects were found for the association between PMIE and posttraumatic growth. In fact, the indirect effect through negative cognitions alone was not statistically significant. Yet, the subsequent effect of negative cognitions and self-care was statistically significant (β = −0.04, *p* = 0.005), meaning that the more aid workers reported PMIE exposure, the more they endorsed negative cognitions which was associated with less self-care, leading to less posttraumatic growth. In addition, the indirect effect through self-care alone (β = 0.07, *p* = 0.03) was also statistically significant, meaning that independently from the changes in negative cognitions, self-care had an additional positive influence on posttraumatic growth. The direct path remained significant for posttraumatic growth (β = 0.23, *p* = 0.001).

Overall, the model explained 52.5% of the variance of PTSD symptoms, 33.2% of depression symptoms, and 21.1% of posttraumatic growth. The fit indicators revealed an excellent adjustment between the data and the final model: *X*^2^ = 1.64, *p* = 0.44; TLI = 1.01; CFI = 1.00; RMSEA < 0.001; 90% CI (0.00, 0.12); SRMR = 0.02.

## 4. Discussion

There had been no study on PMIE in aid workers or on how such events were related to their mental health. Further, even in populations where PMIE had been substantially studied, such as military populations, only a handful of studies had examined how PMIE related to *positive* or growth-related mental health indicators. Finally, there was limited evidence pertaining to the mechanism that might be at play in explaining the relationship between PMIE and mental health indicators. Therefore, the current study sought to examine the presence of PMIE in aid workers, and to investigate how PMIE were associated to PTSD symptoms, depression symptoms and posttraumatic growth. The second goal of this study was to examine if negative cognitions and self-care could play a role in the relationship between PMIE and PTSD symptoms, depression symptoms, and posttraumatic growth, above and beyond the contribution of potentially traumatic events.

Several salient findings of this study are worth discussing. First, experiencing PMIE in the context of aid work appears to be the norm rather than the exception, as 81.1% aid workers reported PMIE. Precisely, 22.8% reported having committed a PMIE, 75.3% reported having witnessed others committing a PMIE, and 27.4% reported PMIE that stemmed from being betrayed. Thus, our results confirm that aid workers experience high rates of PMIE, as has been found in an increasing number of high-risk occupational groups (Williamson et al., 2018). For comparison, one study of deployed armed forces reported that 65.2% were exposed to PMIE (Hansen et al., 2021). Another study suggested that approximately 10.8% of combat veterans reported having committed PMIE, 25.5% reported having witnessed others committing PMIE, and 25.5% endorsed betrayal (Wisco et al., 2017). Therefore, our prevalence of PMIE appears to be somewhat higher than that of other populations. It is important to note that, until recently, measures of PMIE for non-military populations were adapted from those developed specifically in the context of military service (e.g., Hoffman et al., 2018). Because aid workers do not have the same mandate (e.g., must remain neutral in conflicts; relieve the local population by enduring often painful social conditions) or chain of command/problem-solving structure, we believe that they may be more vulnerable to different experiences of PMIE than their military counterparts. For instance, aid workers may have to tolerate more morally unacceptable situations or witness unjust acts in their day-to-day work than military personnel. They may also be more often exposed to situations where they feel powerless, they cannot take an open stand, or are unable to implement solutions quickly to resolve injustices or difficult circumstances. Therefore, it is possible that our high incidence of PMIE, especially of PMIE relating to others’ actions (75.3%), could encompass different types of PMIE. Future studies of aid workers should investigate nuances in the types of PMIE, specifically those that could relate to having chosen to not act in a situation where they feel that they morally should have, having been constrained to not act in the face of wrongdoing or repeatedly witnessing human suffering toward which there is no immediate solution (e.g., Chaplo et al., 2019). Furthermore, future studies could investigate in more detail the types of betrayals (e.g., by a colleague, an organization) that aid workers’ experience. Our results are important as they provide an initial picture of the types of PMIE that aid workers’ experience. They also highlight that identifying with more detail what PMIE are most common or harmful will be the next step to reduce their incidence in the field or to mitigate their consequences.

Another avenue to explore to explain the high prevalence of PMIE would be to examine the expectations of aid workers and how prepared they are for the moral challenges of their work. Indeed, as PMIE results from the perceived gap between one’s moral expectations and the perceived morality of events, investigating ways of reducing this gap could be fruitful. Some research has found that aid workers often embrace an altruistic identity (McCormack et al., 2009; McCormack and Joseph, 2012) which may lead to expectations that they will “do good.” Aid workers may be trained and mentally prepared to face threats to their lives or physical safety. However, they may be much less prepared to witness deliberately unjust or inhumane acts, or to have to comply with restrictions that seem contrary to what is legitimate, right, or logical. This lack of preparedness may add to the inconceivability of PMIE, and perhaps contribute to the traumatic impact of such events. A qualitative study conducted with British military personnel sought to understand the factors that personnel perceived as contributing to the development of moral injury (Williamson et al., 2020). This study identified that a perception of not being warned or prepared for the emotional and psychological consequences of morally difficult decisions or situations was associated with the development of moral injury (Williamson et al., 2020). Some studies are beginning to explore whether mental preparation for morally challenging situations might have a protective effect against moral injury (e.g., Bowman et al., 2021). In our view, an important avenue of research would be to better understand aid workers’ moral expectations and to examine whether increasing aid workers’ psychological preparedness for the moral challenges of their occupation could be beneficial. The results of this type of study would provide valuable avenues for helping aid workers cope with PMIE and for preventing PMIE from leading to sustained distress.

Results from the correlation matrix also provide interesting findings. The positive associations between PMIE and PTSD and depression symptoms have been amply studied in military populations [see systematic reviews by McEwen et al. (2021) and Williamson et al. (2018)] and were replicated in this study. However, the positive association of PMIE with posttraumatic growth provides additional support to the idea that growth can cohabit with distress in the context of PMIE. In fact, this implies that aid workers can experience positive life changes relating to the morally challenging situations they encounter, while also bearing significant psychological distress.

Likewise, our main serial structural equation model has important implications. One key finding is that negative cognitions appear to contribute to the development of psychological distress in the face of PMIE. In fact, the associations between PMIE and symptoms of PTSD and depression disappeared once the indirect path via negative cognitions was included. Consistent with models of psychological adaptation to PMIE (Litz et al., 2009), our findings support how cognitions have a central role in post-PMIE psychological adaptation (Held et al., 2017). This finding also provides additional support for interventions for PMIE-related distress that target negative cognitions (Griffin et al., 2019; Currier et al., 2021). In fact, several studies have found that intervention protocols that target negative cognitions are effective at reducing PTSD and depression symptoms in morally injured veterans (e.g., Griffin et al., 2019). For aid workers, we could thus consider interventions that specifically target how they perceive morally injurious events by recontextualizing negative thinking (that is, providing context to morally injurious events while acknowledging appropriate responsibility; not viewing thoughts as erroneous but as arising from an inflated sense of responsibility) or interventions that aim to change how they interact with these thoughts (e.g., cognitive defusion; Currier et al., 2021).

Interestingly, we also found that negative cognitions contributed to dampening the positive outcomes related to PMIE. Specifically, negative cognitions were associated with a decrease in self-care which contributed to lower posttraumatic growth. It should be emphasized that negative cognitions only negatively contributed to posttraumatic growth when associated with subsequent reductions in self-care. This aligns with theoretical models of posttraumatic growth (Tedeschi and Calhoun, 2004; Joseph, 2012). In fact, in attempting to reduce the gap between previously held beliefs and the meaning of the traumatic event, trauma exposed individuals will engage in cognitive processing aimed at making sense of the trauma, which will generate emotions and related coping behaviors (Tedeschi and Calhoun, 2004; Joseph, 2012). In doing so, individuals may reassess their life goals, relationships, and sense of personal strength or faith and implement behaviors that are aligned with these cognitions, such as self-care behaviors, leading to posttraumatic growth (Tedeschi and Calhoun, 2004; Joseph, 2012). Therefore, by finding that negative cognitions only impede growth when they lead to changes in behavior (i.e., self-care behaviors), our results further support that the meaning given to the trauma and the subsequent related coping strategies are central in the amount of posttraumatic growth that will ensue. This provides several interesting avenues for future research. Indeed, one of them is examining how shame or guilt in the context of PMIE might complicate or impede posttraumatic growth. Shame is a painful emotion that is characterized by a feeling of “being small,” unworthy, and underserving (Currier et al., 2021, p. 41) and that motivates behaviors such as social withdrawal, self-isolation, and self-punitive or self-deprecating behaviors (e.g., Elison et al., 2006). Therefore, when aid workers have negative perceptions (e.g., of themselves) after having experienced PMIE, they also tend to withdraw from self-care, which could be indicative of strong feelings of shame (Lee et al., 2001). Another area of research to consider is investigating *how* aid workers think about the PMIE that they experience rather than *what* they think about. Given that the cognitive work and processing that occurs after trauma is essential to growth (Tedeschi and Calhoun, 2004; Joseph, 2012), cognitive processes (e.g., rumination; Bravo et al., 2020) rather than cognitive content could also be important in promoting posttraumatic growth after a PMIE. Future studies could investigate how cognitive content and processes might interact to influence psychological adaptation to PMIE.

Another key finding is the importance of self-care in promoting posttraumatic growth. Indeed, on top of the indirect effect found via negative cognitions and consequent changes in self-care, a significant indirect effect emerged where PMIE was directly associated with more self-care and more posttraumatic growth. Therefore, aid workers who experienced more PMIE and tended to increase their self-care showed more posttraumatic growth. Encouraging aid workers to maintain or increase self-care even in difficult circumstances could be a way to promote growth, although this should be further investigated. We could therefore propose that aid workers become even more involved in activities that promote their vitality in all aspects of their wellbeing, professional, emotional, physical, spiritual, and psychological. For instance, helping them recognize the importance of maintaining these strategies (or finding forms of self-care that are doable) even when they are overwhelmed, anxious, stressed, or depressed could important as it has been found to promote long-term psychological adjustment in other professional populations (Brillon et al., 2023).

Somewhat surprisingly, self-care (or lack thereof) did not significantly contribute to explaining PTSD and depression symptoms. In fact, as self-care can often be a form of rewarding or enjoyable activity, one could expect that a decrease in self-care could contribute to increased PTSD and depression. However, we did not find this in our study. Some of the key features of moral injury are a sense of social disconnection and the idea that one’s perception of a “shared moral code” has been fractured by the PMIE (Griffin et al., 2019). Therefore, this result could reflect that self-care, which often implies solitary activities (e.g., sleeping enough, taking breaks during the workday) as opposed to social ones, could do little in reversing these features that lead to enduring distress (i.e., symptoms of PTSD and depression). Engaging in activities that promote social connection or seeking social support could be more important in preventing distress, whereas self-care could contribute more strongly to positive aspects of mental health. For instance, some studies of military personnel have suggested that working in a highly cohesive team may be protective against moral injury (e.g., Bryan et al., 2018). As aid workers often live and work in group settings, examining whether team cohesiveness or a sense of connectedness might play a role in how aid workers adapt psychologically to PMIE appears like a promising next step in research. Future studies should also seek to know more about how self-care relates to other positive mental health indicators such as wellbeing or quality of life. Nonetheless, these results are important as, until now, self-care had not been investigated in the context of PMIE. In fact, the emergent but limited literature of mediators or moderators of the association between PMIE and mental health indicators appear to have mainly looked at cognitive (e.g., meaning making; Held et al., 2017; Mordeno et al., 2022), emotional (e.g., emotional regulation; Ray et al., 2021) or social (e.g., interpersonal needs; Ray et al., 2021) mechanisms, thereby overlooking behavioral mechanisms. Yet, behavioral mechanisms are interesting to study as they are arguably easily implementable prevention measures and can also be integrated into prevention and intervention protocols.

### 4.1. Limitations

The current study is not without limitations. First, because the study used a cross-sectional design, the directionality and causal nature of the associations found remain undetermined and should be studied using longitudinal designs. Second, the assessments of PMIE and mental health indicators were obtained using self-report measures, which can introduce bias such as recall or social desirability. Future studies should favor more objective methods, such as clinician-rated assessments. Third, because our recruitment was done via organizations, we were not able to know how many aid workers were contacted and to estimate response rates. Fourth, the sample originated and worked all over the globe, but cultural factors were not directly investigated. Given the cultural influences on individuals’ beliefs about morality, right and wrong, and personal goodness, it would be important to examine how cultural norms and spiritual beliefs may influence individuals’ experiences of and reactions to PMIE. Considering how professional culture or identity may be related to PMIE could also be important (e.g., altruistic identity; McCormack and Joseph, 2012). Fifth, while we did control for lifetime exposure to potentially traumatic events, we believe that considering how additional personal factors (e.g., complex trauma, cognitive flexibility) could contribute to PMIE’s detrimental psychological effects would be important. Lastly, this study did not consider other potential mediators or moderators that could add to the explanation of associations between PMIE and mental health indicators (e.g., social support, role of personal values). Additional studies are needed to further explore the complex processes by which PMIE may impact aid workers’ mental health. Despite these limitations, this study brings attention to an important risk factor for aid workers’ mental health, PMIE, and sheds new light on two mechanisms by which PMIE might lead to PTSD, depression, and posttraumatic growth, that is negative cognitions and self-care.

### 4.2. Implications

This research offers some insight into the PMIE that aid workers experience and into the factors that contribute to their associations with mental health outcomes. Therefore, results provide precious cues to preventing PMIE from leading to enduring distress, and to helping aid workers cope with PMIE. First, given the pervasiveness of PMIE, it could be helpful to provide psychoeducation to aid workers during initial training or during their transition out of an assignment. This psychoeducation would aim to explain that PMIE will unfortunately be common in their assignments and to give examples of the most frequently encountered PMIE in aid work to increase preparedness and cognitive flexibility in the face of moral challenges. Normalizing that aid workers may struggle to accept some of their experiences, others’ actions, or even their own choices would be important, and could appease some of the shame and guilt related to these events. Furthermore, it would be important to normalize that those who experience PMIE often struggle with shame, guilt and anger (Griffin et al., 2019; Yeterian et al., 2019), and that these emotions may motivate them to withdraw, to push others away or to feel that they should be punished. This could encourage aid workers to disclose PMIE, seek support from peers, or show more self-compassion, all of which could help prevent PMIE from leading to more enduring psychological distress. This psychoeducation would also be an opportunity to better equip aid workers with some of the skills that may need to adaptively resolve inner conflict after a PMIE. This would be an opportunity to provide ways of coping with PMIE and the painful cognitions and emotions that may arise (e.g., cognitive defusion, challenging their thinking, practicing self-compassion or distress tolerance, seeking out positive social support). Aid organizations could also provide guidance on practicing self-care (e.g., expanding the notion of self-care, giving ideas of self-care in all domains, realistic ways making self-care a priority). For instance, promoting self-care daily, but even more so in situations of PMIE, appears to be a promising avenue to promote psychological growth. Additionally, providing training to aid workers in leadership roles about PMIE and about the signs of moral injury to look out for in colleagues could also be a worthwhile endeavor. Lastly, mental health professionals who assist aid workers should consider PMIE in their initial assessments and open the discussion about PMIE, as disclosure of PMIE may be challenging for many.

This study also has important empirical and theoretical implications. For instance, it suggests that when trying to understand the psychological implications of PMIE, focusing only on psychological distress can mask the potential for growth that can co-occur in relation to PMIE; aid workers may show great resilience while also being distressed by parts of their experiences. Our study also further supports some of the key elements of theoretical models of PMIE (Litz et al., 2009). By investigating self-care as a potential mechanism by which PMIE may influence mental health, we provide support for the behavioral mechanisms suggested in models that had been only scantly investigated. Lastly, our research highlights that aid workers face PMIE and may struggle with moral injury. Understanding what types of PMIE aid workers frequently encounter and how to help these professionals cope with PMIE appear to be fruitful next steps in research.

## Data availability statement

The raw data supporting the conclusions of this article will be made available by the authors, without undue reservation.

## Ethics statement

The studies involving human participants were reviewed and approved by University of Quebec in Montreal Institutional Ethics Committee on Human Research. The participants provided their written informed consent to participate in this study.

## Author contributions

MD conducted the statistical analyses and wrote the initial manuscript. AP and PB assisted in the analyses and reviewed the manuscript. All authors designed and conducted the study (e.g., ethics approval, data collection). All authors contributed to the article and approved the submitted version.
