# Supplementary material for: Morally injurious events among aid workers: examining the indirect effect of negative cognitions and self-care in associations with mental health indicators
Source: Front Psychol. 2023 Apr 13;14:1171629. doi: 10.3389/fpsyg.2023.1171629 (PMC10133712; doi:10.3389/fpsyg.2023.1171629)
Supplement: Supplementary file 1 [file Table_1.docx]

Supplementary Material

**Morally Injurious Events among Aid Workers: Examining the Indirect Effect of Negative Cognitions and Self-Care in Associations with Mental Health Indicators**

**Michelle Dewar^1,2^, Alison Paradis^1^ and Pascale Brillon^2*^**

***Correspondence**: Pascale Brillon : [brillon.pascale@uqam.ca](mailto:brillon.pascale@uqam.ca)

**Supplementary table 1.**

*Degree of PMIE Exposure using MIAS Items*

|  | % | *M* | *SD* |
| --- | --- | --- | --- |
| PMIE related to others’ actions (4 items) | 75.3 | 2.72 | 0.80 |
| *I am troubled by morally wrong things done by other people.* | 67.1 |  |  |
| *I am troubled because other people have acted against important moral rules.* | 57.2 |  |  |
| *I am troubled because I saw other people do things that were morally wrong.* | 51.4 |  |  |
| *I am troubled because I heard about other people doing things that were morally wrong.* | 49.8 |  |  |
| PMIE related to one’s own actions (5 items) | 22.8 | 1.45 | 0.64 |
| *I am troubled because I did things that were morally wrong.* | 12.8 |  |  |
| *I went against my own morals by failing to do something I should have done.* | 12.8 |  |  |
| *I am troubled because I acted in ways that went against my own moral code or values.* | 11.2 |  |  |
| *I am troubled by morally wrong things I have done.* | 8.7 |  |  |
| *I am troubled because I acted against important rules.* | 7.0 |  |  |
| PMIE related to betrayal (2 items) | 27.4 |  |  |
| *I feel betrayed by people I trusted.* | 26.0 | 1.60 | 0.66 |
| *I betrayed people who trusted me.* | 5.4 |  |  |
| All items | 81.1 | 1.94 | 0.53 |

*Note.* PMIE = potentially morally injurious events. Items that were rated 3 and above were considered endorsed. Means are rated on the following scale: 1= not at all, 2 = a little, 3 = a lot, 4 = very much. On average, participants endorsed 3.09 items (SD = 2.44).
